# Changes in Structures and Properties of Collagen Fibers during Collagen Casing Film Manufacturing

**DOI:** 10.3390/foods12091847

**Published:** 2023-04-29

**Authors:** Fei Liu, Zhe Yu, Beibei Wang, Bor-Sen Chiou

**Affiliations:** 1 Key Laboratory of Synthetic and Biological Colloids, Ministry of Education, Jiangnan University, Wuxi 214122, China; yuzhejiji@sina.cn (Z.Y.);; 2 Science Center for Future Foods, Jiangnan University, Wuxi 214122, China; 3 School of Food Science and Technology, Jiangnan University, Wuxi 214122, China; 4 Western Regional Research Center, ARS, U.S. Department of Agriculture, Albany, CA 94710, USA

**Keywords:** collagen casings, collagen fiber, manufacturing process, triple-helix structure

## Abstract

Collagen casing is an edible film, which is widely used in the industrial production of sausages. However, the detailed changes in the collagen fibers, from the raw material to the final collagen film, have rarely been reported. In this research, the changes in the collagen fibers during the manufacturing process, including the fiber arrangement, the triple-helix structure and the thermal stability, were investigated using scanning electron microscopy (SEM), thermogravimetric analysis (TGA), X-ray diffraction (XRD), differential scanning calorimetry (DSC) and Fourier-transform infrared (FTIR) spectroscopy. The relationship between the structure stability and the arrangement of the collagen fibers was also discussed. According to the SEM, XRD, TGA, DSC and FTIR results, the collagen fibers were depolymerized during the acid swelling and became uniformly aligned after the homogenization process. Degassing had no obvious effect on the triple-helix structure. Alkaline neutralization with ammonia destroyed the triple-helix structure, which could be partly reversed through the washing and soaking processes. During the final drying step, the depolymerized triple helix of the collagen fibers recombined to form new structures that showed decreased thermal stability. This study expands our knowledge about the behavior of collagen fibers during the industrial process of producing collagen biobased casings.

## 1. Introduction

Artificial casings are widely used in the meat industry [1,2] due to the high cost and the insufficient supply of natural casings. Among them, collagen casings have gained popularity in the meat industry due to their edibility and their similar taste to natural casings [3,4]. Many studies showed that collagen casings not only provided a coating for the meat but also affected the quality of the sausages. Some researchers demonstrated that collagen casings could improve the quality characteristics of the sausages, when compared to pork casings, by reducing the biogenic amines produced during fermentation [5]. Another advantage of collagen casings is the ease with which additional ingredients can be added to confer functionality, such as antimicrobial properties [6].

The characteristics of collagen casings are affected by other materials added during the casing manufacturing process. Collagen, macromolecular polysaccharides such as cellulose, or small molecular compounds with aldehyde groups could interact through hydrogen or covalent bonding, which resulted in changes in the casing properties [7,8]. The addition of various food polysaccharides, such as cellulose, high-amylose starch, hydroxypropyl methylcellulose and guar bean gum of different molecular weights, could also affect the performance of the collagen casings [9]. Some researchers also reported that the use of fumigate, including aldehyde compound and chitosan, improved the mechanical properties of collagen casings [10,11].

The microstructure, the arrangement of the collagen fibers and the cross-linking between the collagen fibrils also affected the mechanical properties of the collagen tissues. Collagen casings have natural long-fiber frameworks, high-aspect-ratio fibers and cross-linking in vivo that lead to a high Young’s modulus and strong biological tissues [12,13]. Previous research showed that the change in collagen fiber alignment caused by the controlled counter-rotating cone extrusion (typical of the treatment used in collagen casing production) significantly changed the elasticity and the toughness of the film [14]. A similar phenomenon was observed in the bioprosthetic aortic valve composed of collagen [15].

Some physicochemical treatments during the manufacture of the collagen casing could also improve the film’s properties due to the changes in the fiber microstructure. Studies showed that thermal dehydration treatment could increase the cross-linking density of the collagen fibers and lead to an improvement in the mechanical properties of the collagen casings [16]. PH and temperature could also affect the performance of the films by modifying the layered fiber network and the triple-helix contents [17,18]. These studies further demonstrated the influence of the collagen fiber structure on the film properties.

Collagen casing is usually extracted from animal skin and processed through multiple steps, such as acid swelling, neutralization, plasticization and hot air drying [19]. These processes, from the fiber extraction of the raw materials to the recombination of the films, inevitably alter the collagen structure and arrangement. However, the changes in the collagen fibers during the traditional collagen casing process have not been studied in detail, and the relationship between the collagen fiber structure and the casing performance is not clear.

In this study, cowhide skin was used as a raw material to produce collagen casing films. The changes in the microstructure of the collagen fibers at different processing stages during the artificial casings’ preparation were studied. The results obtained can help us to understand the changes in the collagen from the cowhide to the final collagen casing film and thus provide a basis to improve the quality of artificial collagen casings.

## 2. Materials and Methods

### 2.1. Materials and Reagents

The cowhide was provided by Beijing Qiushi Agriculture Development Co., Ltd. (Beijing, China). The carboxymethylcellulose sodium was purchased from Chongqing Lihong Fine Chemical Co., Ltd. (Chongqing, China). The hydrochloric acid and aqueous ammonia were of analytical grade and obtained from Sinopharm Chemical Reagent Co., Ltd. (Shanghai, China).

### 2.2. Preparation Process of Collagen Casing Films and Sampling

The collagen casings were prepared according to a previous study [20] and a patent of an industrial manufacturing process [19]. First, the desalted cowhide was cut into small pieces and ground in a high-speed pulverizer. Crushed ice was added to keep the temperature below 20 °C, and a solid content of 9.5–10% was obtained. Then, 25 g of the wet cowhide slurry was mixed with a 0.5% hydrochloric acid solution (calculated as pure hydrochloric acid) and acidified to expand the sample at 4 °C for 18–24 h. The swelled collagen was homogenized under a pressure of 325 bar and then degassed using a vacuum at 0.8 MPa. The degassed collagen was extruded to form films and then soaked in a 5% ammonia solution (calculated as pure ammonia gas) for 1 min to neutralize the hydrochloric acid. These films were washed with deionized water until they became neutral, dried at 75 °C for 4 h and then placed in a self-sealing bag.

**Figure 1:**
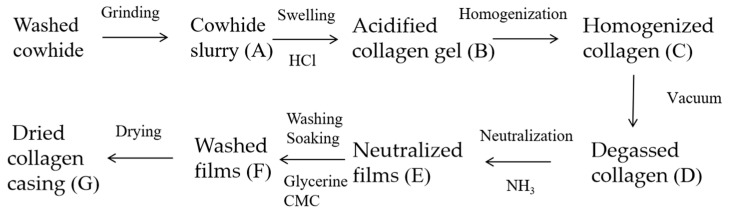
Manufacturing process of collagen casing films.

The process is shown in Figure 1. The cowhide slurry, acidified collagen, homogenized collagen, degassed collagen, neutralized films, washed films and dried collagen casing films obtained in each step were saved for subsequent studies. These samples were frozen at −80 °C for 2 h and then freeze-dried for 24 h.

### 2.3. Characterization of Collagen Fibers and Films

#### 2.3.1. Moisture Content Test

The cowhide slurry, the neutralized film and the washed film were treated using the following method before conducting the water content test.

The samples were wrapped with sufficient absorbent paper and placed on the press machine (JSP-600HA, Shanghai Jingsheng, Shanghai, China). A pressure of 5 MPa was applied to the samples and maintained for 5 min.

The acidified, homogenized and degassed collagen gel could be directly used to determine the moisture content. The dried casing film was equilibrated in a chamber with a constant temperature and relative humidity of 25 °C and 53% for 24 h prior to testing. The abovementioned samples were then dried in a 105 °C oven until a constant weight was reached, and then the moisture content was calculated.

(1) $$Moisture\;content\;\left(\%\right)=\left(1-\frac{m-m_{0}}{m}\right)\times 100$$

*m* is the initial weight of each sample, and *m*_0_ is the dried constant weight of each sample.

#### 2.3.2. Mechanical Property Test

Commercial casing samples were longitudinally cut into strips of 10 mm × 50 mm, and their tensile properties were measured using a texture analyzer (TA. XT2i, Lloyd Instruments, Bognor Regis, UK).

The neutralized film, washed film and dried film were also cut into strips of 10 mm × 50 mm for testing.

The testing conditions were as follows: fixture: A-MTG; trigger force: 10 g; pre-test speed: 0.5 mm/s; test speed: 0.5 mm/s; post-test speed: 0.5 mm/s.

The tensile strength (TS) of the samples was calculated using the following equation:

(2) $$TS=Stress=\frac{F}{Thickness\times width}$$

The thickness was the average thickness of the collagen film measured at 5 points, and the width of each sample was 10 mm. *F* was the force applied to stretch the film. The strain was calculated using the following equation:

(3) $$Strain\left(\%\right)=\frac{L-L_{0}}{L_{0}}\times 100$$

*L* is the length of the film when it broke, *L*_0_ is the initial length of the film.

#### 2.3.3. Scanning Electron Microscopy (SEM) Analysis

The freeze-dried samples described in Section 2.2 were fractured in liquid nitrogen. The sample surface was coated with gold, and the sample’s cross-section structure was observed using a scanning electron microscope (Quanta 200, FEI, Hillsboro, OR, USA). The accelerating voltage was 3.0 kV.

#### 2.3.4. Fourier-Transform Infrared (FTIR) Spectroscopy Analysis

The freeze-dried collagen slurry and fibrils were pressed into thin films, and the collagen casing films were cut into small pieces. The samples were then conditioned at 53% relative humidity (RH) and 25 °C for 72 h. The chemical functional groups in the samples were determined using a Fourier-transform infrared spectrometer (Nicolet IS 10, Thermo Electron, Waltham, MA, USA). Each sample was scanned 32 times from 4000 to 400 cm^−1^ at a resolution of 8 cm^−1^ [21].

#### 2.3.5. Differential Scanning Calorimetry (DSC) Analysis

The samples were cut into small pieces of 2 × 2 cm and then conditioned in an acrylic drying box at 53% RH (25 °C) for 72 h. The thermal properties of the samples were determined using a differential scanning calorimeter (Discovery DSC25, TA, New Castle, DE, USA). Each sample was heated from 25 to 150 °C at 5 °C/min.

#### 2.3.6. Thermogravimetric (TGA) Analysis

The samples were first conditioned at 53% RH (25 °C) for 72 h. The thermal stability of the samples was determined using a TGA (1100SF, METTLER TOLEDO, Greifensee, Switzerland). Each sample was heated from 25 to 600 °C at a heating rate of 10 °C/min.

#### 2.3.7. X-ray Diffraction (XRD) Analysis

The samples were first conditioned at 53% RH (25 °C) for 72 h. The crystallinity of the samples was determined using an X-ray diffractometer (D2 Phaser, Bruker AXS, Karlsruhe, Germany). Each sample was scanned from 5 to 50° using a step width of 0.02° and a scanning rate of 0.02°/0.1 s.

### 2.4. Data Analysis

The infrared spectra were processed using OMNIC software. The data from the differential calorimetry (DSC) analysis, the thermogravimetric (TGA) analysis and the X-ray diffraction (XRD) analysis were analyzed and graphed using Origin 2022 software. The significant differences between the mean values (*p* < 0.05) were determined through Duncan’s multiple range test using SPSS 26 software.

## 3. Results and Discussion

### 3.1. Moisture Content and Mechanical Properties

**Figure 2:**
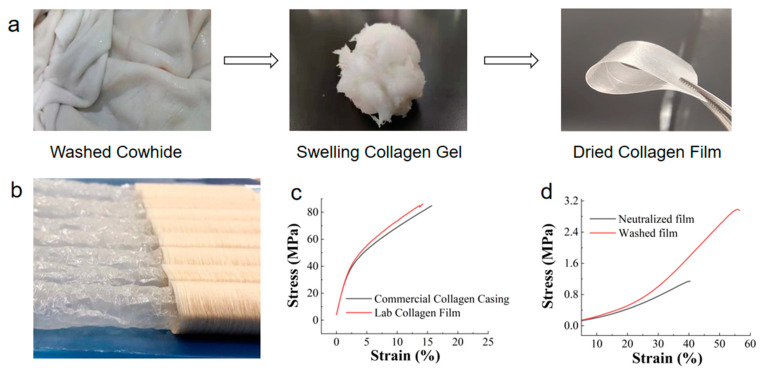
(**a**) A lab-scale fabrication from cowhide material to collagen fiber films. (**b**) Commercial collagen casings. (**c**) Stress–strain curves of the commercial collagen casing and the lab-made collagen film. (**d**) Stress–strain curves of the neutralized film and the washed film.

It is well known that collagen casing is formed through the processes of collagen fiber hydration, extrusion molding and dehydration, which represent the remodeling process of collagen fibers. Table 1 lists the collagen moisture contents during the various stages of laboratory preparation. The principles and processes of laboratory preparation were similar to those in industrial production. Figure 2a shows the images of the cowhide skin, the swollen collagen gel and the final casing film in laboratory-scale preparation.

Initially, the ordered, dense and high-strength collagen fibers were assembled into fiber bundles from the cowhide tissue. The collagen bundles were disrupted after cutting. When the hydrochloric acid was added, the side chain groups of the collagen peptides in the fiber bundle, which carry positive charges, led to electrostatic repulsion, increasing the distance between the collagen peptide chains. At the same time, water permeated into the fiber bundles, causing them to swell, or even to disintegrate, which resulted in a swelling collagen gel, as shown in Figure 2a. The collagen gel had a certain deformability and fluidity. The moisture content of the gel was 93.3–93.5%.

The neutralization process resulted in deswelling, reducing the moisture content from 93.5% to 84.5%. Finally, a film with a moisture content of 19.5% was obtained after controlled hot air drying. The neutralization and drying process caused dehydration of the collagen fibers. As the water evaporated, the intermolecular spacing decreased and the interaction between the molecules was enhanced. The swelling collagen fibers underwent self-assembly and alignment to ultimately form a network of the film.

The mechanical property testing was conducted on the neutralized film, washed film and dried collagen film, as shown in Figure 2c,d. The collagen gel swollen by the hydrochloric acid could be molded into any shape. After being pressed into a thin film, it was then fixed with ammonia water to obtain a wet film. However, it is worth noting that the mechanical strength of the neutralized films was only 1.1 MPa, indicating that the collagen fibers did not form a strong network structure in this stage, resulting in low strength. Interestingly, after washing and soaking treatments, the mechanical strength of the water-washed films was significantly improved compared to the neutralized films, reaching 2.9 MPa. The relationship between this improvement and the collagen structure will be discussed in later sections.

The mechanical strength of the dried sausage casing film was greatly increased, reaching 86.0 MPa, which could be attributed to the dehydration and assembly of the collagen after drying, leading to a strong network. The commercial sausage casings were purchased from the market (Figure 2b). The tensile strength of the commercial casings was 84.7 MPa, similar to the laboratory-made collagen casing film. This indicated the successful simulation of the industrial process in the laboratory. It is worth noting that the solid content of the final film is a key factor affecting its performance. The film needed to maintain a certain moisture content to ensure its flexibility and high elongation at break. In this study, the moisture content of the final film sample was 19.5%. To validate this, the moisture content of commercially available collagen casings was measured; it varied between 17.5% and 21.9%, indicating that the final collagen film obtained in this study had an appropriate moisture content compared with the commercial collagen casings.

### 3.2. Microstructure Characterization of Collagen Fibers

#### 3.2.1. SEM Analysis of Collagen Fibers

**Figure 3:**
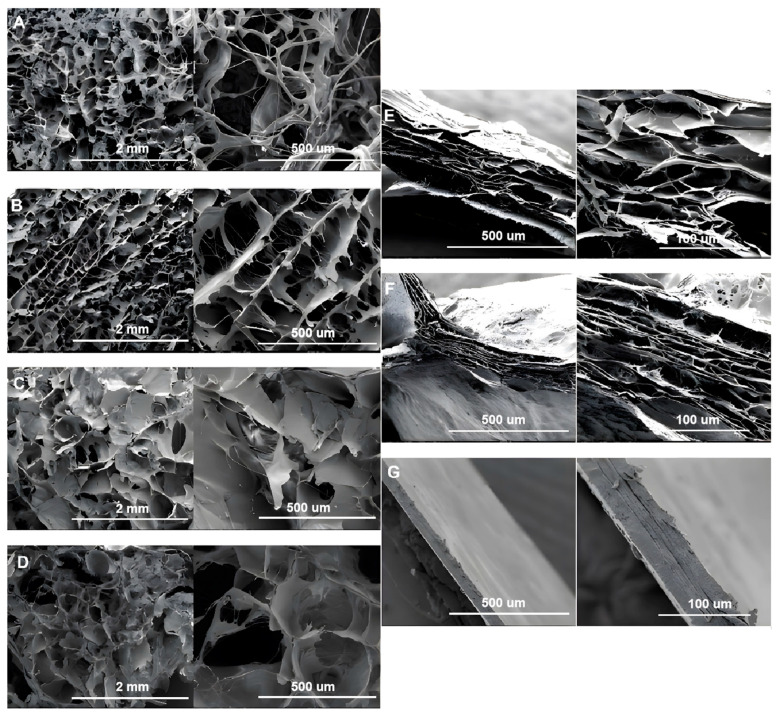
Scanning electron micrographs of the collagen fibers at different stages. The processing stages are (**A**) cowhide slurry, (**B**) acidified collagen, (**C**) homogenized collagen, (**D**) degassed collagen, (**E**) neutralized films, (**F**) washed films and (**G**) dried collagen casing films. The left-side pictures in (**A**–**D**) were magnified 40 times, and the right-side pictures were magnified 160 times. The left-side pictures in (**E**–**G**) were magnified 160 times and the right-side pictures were magnified 600 times.

As shown in Figure 3, the collagen fibers of the freeze-dried slurry and the acid-swollen gels in different processing steps (Figure 3A–D) showed 3D porous network structures. The thick collagen fiber bundles could easily be observed in the cowhide slurry. The acid-swollen gel (Figure 3B) showed the collagen fibers arranged in pieces, with the individual collagen fibers being much thinner and rarer than those in the cowhide slurry. This indicated that the collagen fibers absorbed water and swelled to form finer fibrils during the acid swelling.

The collagen samples, after film formation (Figure 3E–G), showed layered structures with significantly increased tightness due to the dehydration process. The collagen fibers of the dried films were tightly connected, with almost no gap between these layers.

#### 3.2.2. Fourier-Transform Infrared (FTIR) Spectroscopy Analysis of Collagen Fibers

**Figure 4:**
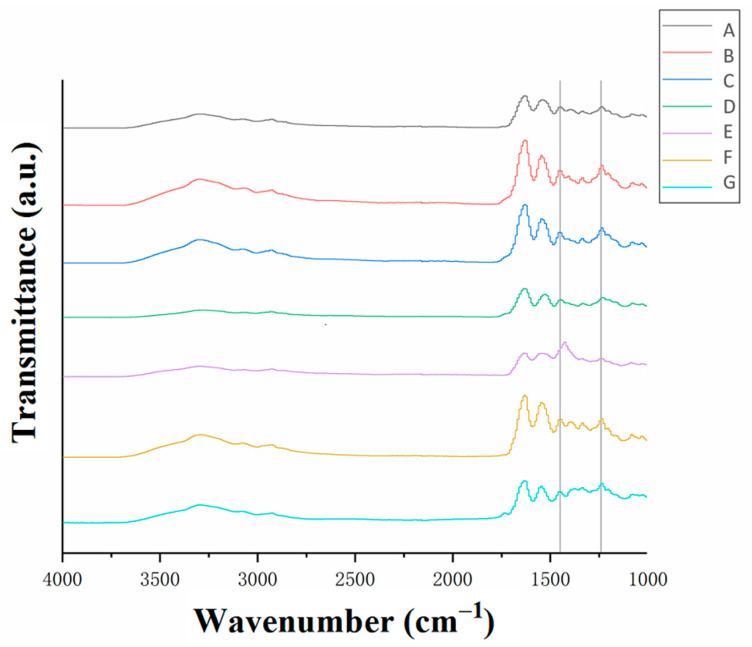
Infrared spectra of the collagen at different treatment stages. The processing stages corresponded to (A) cowhide slurry, (B) acidified collagen, (C) homogenized collagen, (D) degassed collagen, (E) neutralized films, (F) washed films and (G) dried collagen casing films.

Fourier-transform infrared (FTIR) spectra of the collagen samples at different stages during the casing manufacturing process are shown in Figure 4. All the samples exhibited the amide III band in the region of 1500–400 cm^−1^ (the single-bond region). This band was attributed to the stretching vibration of the C-N bond, the bending vibration of the N-H bond and the wagging vibration of the CH2 group in the glycine backbone and proline side chain. The peak absorbance ratio at 1240 cm^−1^ and 1450 cm^−1^ corresponded to the triple-helical structure of collagen [7]. The A_1240_/A_1450_ ratios of each sample are shown in Table 1. The peak absorbance ratio corresponded to the relative amount of the complete triple-helical structure of the collagen, with a higher ratio corresponding to a higher amount [22].

As shown in Table 1, only the neutralized films (Table 2 (row E)), after being treated with ammonia, had a peak absorbance ratio lower than 1. This could be due to the destruction of the triple-helix structure of the collagen caused by soaking it in the basic solution. The destruction could also be due to the formation of salts from hydrochloric acid and ammonia, which led to the denaturation of the collagen and affected the triple-helix aggregation. The subsequent washing and plasticizing steps (Table 2 (row F)) resulted in a more compact arrangement of the collagen fibers. The disassembled triple-helix structures reassembled during the drying process (Table 2 (row G)), resulting in the recovery of the A_1240_/A_1450_ value. This result was consistent with that shown in Figure 3G, where the collagen fibers ultimately formed a densely layered film with an ordered arrangement after drying.

#### 3.2.3. Crystal Structure Analysis of Collagen Fibers

**Figure 5:**
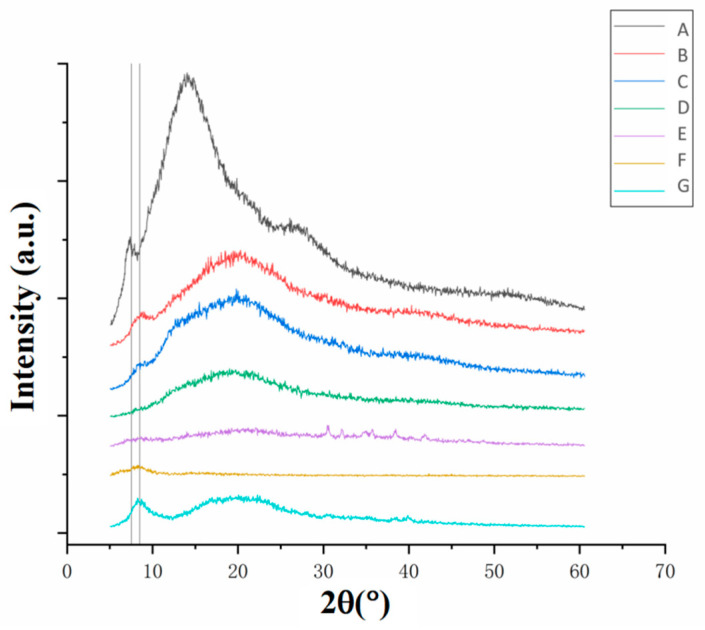
XRD results of the collagen fibers at different stages. The processing stages corresponded to (A) cowhide slurry, (B) acidified collagen, (C) homogenized collagen, (D) degassed collagen, (E) neutralized films, (F) washed films and (G) dried collagen casing films.

A characteristic diffraction peak appeared at 2θ of 7.5° for the cowhide slurry (Figure 5A) and at 8.5° for the other samples (Figure 5B–G). These peaks corresponded to the diameter of the triple-helices of the collagen [23,24]. The peak positions for the B–G curves shifted to higher values compared with the A curve, indicating that the distance between each α-chain in the fiber helix of the collagen increased in value during the acid swelling. The increased distance between the chains was not due to the water absorption by the collagen fibers but to the changes in molecular structure. This is because the peak value of the dried film (Figure 5G) was the same as that of the previous steps. Moreover, the samples showed different peaks at higher 2θ values, indicating changes in crystallinity. The samples obtained in all the subsequent steps after the acid swelling (Figure 5B–G) showed decreases in crystallinity compared with the raw slurry (Figure 5A). In particular, the neutralized films (Figure 5E) showed a significant decrease in crystallinity, which indicated that the ammonia used during the neutralization process damaged the microstructure of the collagen. This was consistent with the FTIR results shown in Table 2. In comparison, the dried film (Figure 5G) showed a sharp increase in crystallinity, which indicated the formation of collagen structures and was also consistent with the FTIR results (Table 2).

### 3.3. Thermal Stability Analysis of Collagen Fibers

#### 3.3.1. TGA Analysis

**Figure 6:**
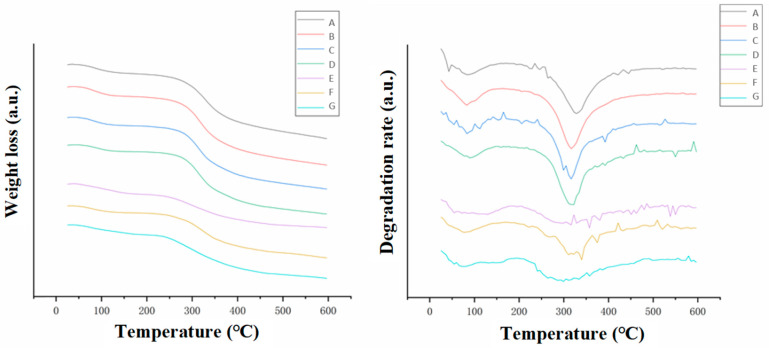
TGA results of the collagen fibers at different stages. The processing stages corresponded to (A) cowhide slurry, (B) acidified collagen, (C) homogenized collagen, (D) degassed collagen, (E) neutralized films, (F) washed films and (G) dried collagen casing films.

The second peak (at 300–330 °C) of the derivative TGA curves in Figure 6 represented the thermal decomposition temperature of the collagen. The decomposition temperatures of the collagen in each step are listed in Table 3. The thermal decomposition temperatures gradually decreased in value from 330 °C to 315 °C after the acid swelling, the homogenization and the degassing (Table 3 (rows A–D)). This indicated a gradual decrease in thermal stability, which was consistent with the damage of the crystalline structure of the collagen fibers during the preparation process (Figure 5). Previous results indicated that alkali neutralization damaged the crystalline structure and the triple-helix integrity of the collagen fibers. However, the thermal decomposition temperature of the neutralized film (Table 3 (row E)) was not significantly different from that of the degassed collagen gel in the previous step (Table 3 (row D)). Moreover, the dried film had the lowest thermal decomposition temperature of 300 °C. This indicated that the high-temperature (75 °C) treatment during the drying process partly destroyed the microstructure of the collagen fibers in the film even though the triple-helix structures were formed and the crystallinity recovered after drying. Apparently, the collagen fibers in the dried film re-aggregated into new triple-helix structures rather than reforming the original one.

#### 3.3.2. Differential Scanning Calorimetry (DSC) Analysis

**Figure 7:**
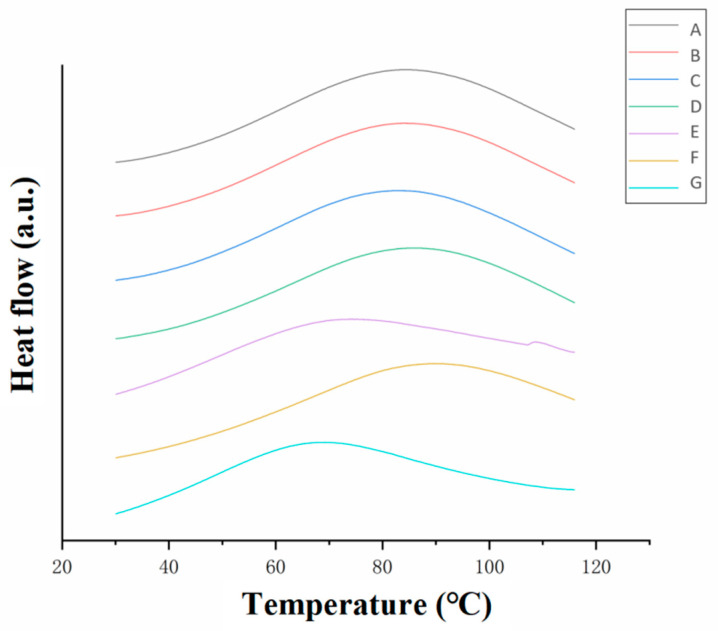
DSC thermograms of the collagen fibers at different stages. The processing stages corresponded to (A) cowhide slurry, (B) acidified collagen, (C) homogenized collagen, (D) degassed collagen, (E) neutralized films, (F) washed films and (G) dried collagen casing films.

The peaks in the DSC curves (Figure 7) corresponded to the thermal denaturation temperature of the collagen and reflected the stability of the collagen fiber structure. Moreover, the peak area in the DSC curves represented the enthalpy value of collagen thermal denaturation. This measured the degree of heat absorption during the denaturation process and directly reflected the thermal stability of the collagen microstructure. A higher enthalpy value of collagen thermal denaturation corresponded to a more stable triple-helix structure of the collagen fiber and increased thermal stability [25,26]. The enthalpy values of collagen denaturation of the samples are shown in Table 4.

The thermal denaturation temperatures of the cowhide slurry, acidified collagen, homogenized collagen, degassed collagen and washed films (Figure 7A–D,F) were between 80 and 90 °C. In comparison, the thermal denaturation temperatures of neutralized and dried collagen casing films (Figure 7E,G) were around 70 °C. The neutralized and dried films also had lower enthalpy values of 288.4 and 208.7 J/g, respectively, indicating that they had poor thermal stability and destroyed microstructures.

The thermal stability of the collagen decreased after neutralization (E) but increased significantly after the water washing, addition of the plasticizer (glycerin and CMC solution) and soaking (F). This was due to the high pH of the ammonia solution and the formation of salts from the hydrochloric acid and ammonia that caused damage to the triple-helix structures of the collagen fibers. However, this damage was reversible and could be minimized through water washing, which was consistent with the FTIR results (Table 2). Furthermore, the neutral environment and the lower salt concentrations after washing affected the recovery of the triple-helix structure of the collagen fiber and improved the thermal stability of the collagen.

The dried collagen casing had the lowest enthalpy value and the lowest thermal denaturation temperature, indicating that the wet heat process during drying damaged the molecular structure. This indicated that although the triple-helix structure of the collagen casings could reaggregate into an ordered structure during drying, the thermal stability of the new structure was clearly inferior to the natural structure of the raw material. This was consistent with the XRD (Figure 5) and the TGA (Figure 6) results.

## 4. Conclusions

Collagen sausage casing films were prepared according to industrial production processes. The microstructure and stability of the samples in each step were then systematically investigated. SEM micrographs of the freeze-dried samples revealed that the collagen fiber bundles became finer in shape under acid-swelling conditions. Further TGA, DSC, FTIR and XRD analyses showed that the acid treatment led to reduced crystallinity, an increase in the diameter of the α-chains and a decrease in the thermal stability. The high pH and the salts generated by the hydrochloric acid and the ammonia during the neutralization process destroyed the triple-helix structure of the collagen. However, this damage was reversible through the removal of the salts and creation of a neutral environment. During the drying process, the triple-helix structures of the collagen fibers reassembled to form a microstructure that was less thermally stable than that of the raw material.

This study systematically investigated the intermediate products produced during the collagen casing preparation process and clarified the effects of acid, alkali and heat treatments on the collagen fiber structure and stability. However, more research is required to determine the mechanisms of the disruption in the triple-helix structure of the collagen fiber and its reassembly during the process. This research could serve as a reference to optimize the manufacturing process and improve the properties of collagen biobased films.

## Figures and Tables

**Table 1:** The moisture contents of the collagen at different stages.

Stages of Samples		Moisture Content/%
A	Cowhide Slurry	73.5 ± 1.2 ^a^
B	Acidified Collagen Gel	93.3 ± 0.3 ^b^
C	Homogenized Collagen Gel	93.5 ± 0.2 ^b^
D	Degassed Collagen Gel	93.5 ± 0.3 ^b^
E	Neutralized Films	84.5 ± 0.9 ^c^
F	Washed Films	83.0 ± 0.9 ^d^
G	Dried Collagen Films	19.5 ± 0.8 ^e^
Control	Commercial Collagen Casing	19.7 ± 2.2 ^e^

Different lowercase letters indicate significantly different values (*p* < 0.05).

**Table 2:** Fourier-transform infrared peak absorption ratios of 1240 cm^−1^ to 1450 cm^−1^ (A_1240_/A_1450_) of the collagen fibers at different stages.

Stages of Samples		A_1240_	A_1450_	A_1240_/A_1450_
A	Cowhide Slurry	0.1006	0.1005	1.000
B	Acidified Collagen Gel	0.1798	0.1627	1.105
C	Homogenized Collagen Gel	0.1571	0.1432	1.097
D	Degassed Collagen Gel	0.08385	0.07801	1.075
E	Neutralized Films	0.08896	0.1268	0.7017
F	Washed Films	0.1682	0.1676	1.004
G	Dried Collagen Films	0.1779	0.1459	1.220

**Table 3:** The thermal decomposition temperatures of the collagen fibers at different stages.

Stages of Samples		Thermal Decomposition Temperature/°C
A	Cowhide Slurry	330
B	Acidified Collagen Gel	320
C	Homogenized Collagen Gel	318
D	Degassed Collagen Gel	315
E	Neutralized Films	318
F	Washed Films	320
G	Dried Collagen Films	300

**Table 4:** The enthalpy values during the thermal denaturation of the collagen fibers at different stages. Different lowercase letters indicate significantly different values (*p* < 0.05).

Stages of Samples		Enthalpy Value/J/g
A	Cowhide Slurry	392.4 ± 15.3 ^a^
B	Acidified Collagen Gel	302.4 ± 18.5 ^b^
C	Homogenized Collagen Gel	302.8 ± 13.5 ^b^
D	Degassed Collagen Gel	310.8 ± 14.7 ^b^
E	Neutralized Films	288.4 ± 12.1 ^b^
F	Washed Films	329.6 ± 18.1 ^b^
G	Dried Collagen Films	208.7 ± 12.4 ^c^

Different lowercase letters indicate significantly different values (*p* < 0.05).

## Data Availability

Data is unavailable.

## References

[1] MarketLine Company Profile: Viscofan SA. https://www.viscofan.com/investor-relations/financial-information-and-annual-report.

[2] Steinke P.K.W., Foster E.M. (2019). Effect of different artificial casings on the microbial changes in refrigerated liver sausage. J. Food Sci..

[3] Zajac M., Pajak P., Skowyra G. (2021). Characterization of edible collagen casings in comparison with the ovine casing and their effect on sausage quality. J. Sci. Food Agric..

[4] Tian X.J., Zhao K.X., Teng A.G., Li Y., Wang W.H. (2022). A rethinking of collagen as tough biomaterials in meat packaging: Assembly from native to synthetic. Crit. Rev. Food Sci. Nutr..

[5] Yan X., Yang L., Zhang Y., Han W., Duan Y. (2022). Effect of collagen casing on the quality characteristics of fermented sausage. PLoS ONE.

[6] Tantala J., Vangnai K., Rachtanapun P., Rachtanapun C. (2019). Active antimicrobial collagen casing. Ital. J. Food Sci..

[7] Chen C., Liu F., Yu Z., Ma Y., Goff H.D., Zhong F. (2020). Improvement in physicochemical properties of collagen casings by glutaraldehyde cross-linking and drying temperature regulating. Food Chem..

[8] Ding C., Yue C., Su J., Wang H., Yang N., Cheng B. (2022). Effects of oxidized cellulose nanocrystals on the structure and mechanical properties of regenerated collagen fibers. Cellulose.

[9] Sobanwa M. (2008). Redesign of Collagen Casings for High Quality Performance Using Food Grade Polysaccharides. Ph.D. Dissertation.

[10] Adzaly N.Z., Jackson A., Kang I., Almenar E. (2016). Performance of a novel casing made of chitosan under traditional sausage manufacturing conditions. Meat Sci..

[11] Barbut S., Ioi M. (2019). An investigation of the mechanical, microstructural and thermo-mechanical properties of collagen films cross-linked with smoke condensate and glutaraldehyde. Ital. J. Food Sci..

[12] Sorushanova A., Delgado L.M., Wu Z., Shologu N., Kshirsagar A., Raghunath R., Mullen A.M., Bayon Y., Pandit A., Raghunath M. (2019). The Collagen Suprafamily: From Biosynthesis to Advanced Biomaterial Development. Adv. Mater..

[13] Gentleman E., Lay A.N., Dickerson D.A., Nauman E.A., Livesay G.A., Dee K.C. (2003). Mechanical characterization of collagen fibers and scaffolds for tissue engineering. Biomaterials.

[14] Hoogenkamp H.R., Bakker G.J., Wolf L., Suurs P., Dunnewind B., Barbut S., Friedl P., Van Kuppevelt T.H., Daamen W.F. (2015). Directing collagen fibers using counter-rotating cone extrusion. Acta Biomater..

[15] Suárez S., López-Campos J.A., Segade A., Veiga C.G., Jiménez V.A. (2022). An study on the influence of collagen fiber directions in TAVs performance using FEM. J. Mech. Behav. Biomed. Mater..

[16] Chen X., Zhou L., Xu H., Yamamoto M., Shinoda M., Kishimoto M., Tanaka T., Yamane H. (2020). Effect of the Application of a Dehydrothermal Treatment on the Structure and the Mechanical Properties of Collagen Film. Materials.

[17] Xu J., Liu F., Goff H.D., Zhong F. (2020). Effect of pre-treatment temperatures on the film-forming properties of collagen fiber dispersions. Food Hydrocolloid..

[18] Xu J., Liu F., Wang T., Goff H.D., Zhong F. (2020). Fabrication of films with tailored properties by regulating the swelling of collagen fiber through pH adjustment. Food Hydrocoll..

[19] US2005031741 A1 Collagen Casing. https://www.freepatentsonline.com/y2005/0031741.html.

[20] Jingmin W., Fei L., Zhe Y., Yun M., H Douglas G., Jianguo M., Fang Z. (2020). Facile preparation of collagen fiber-glycerol-carboxymethyl cellulose composite film by immersing method. Carbohydr. Polym..

[21] Shi D., Liu F., Yu Z., Chang B., Goff H., Zhong F. (2019). Effect of aging treatment on the physicochemical properties of collagen films. Food Hydrocolloid..

[22] Nilsuwan K., Fusang K., Pripatnanont P., Benjakul S. (2022). Properties and Characteristics of Acid-Soluble Collagen from Salmon Skin Defatted with the Aid of Ultrasonication. Fishes.

[23] Badii F., MacNaughtan W., Mitchell J.R., Farhat I.A. (2014). The Effect of Drying Temperature on Physical Properties of Thin Gelatin Films. Dry Technol..

[24] Cheng S., Wang W., Li Y., Gao G., Zhang K., Zhou J., Wu Z. (2019). Cross-linking and film-forming properties of transglutaminase-modified collagen fibers tailored by denaturation temperature. Food Chem..

[25] Persikov A.V., Xu Y., Brodsky B. (2004). Equilibrium thermal transitions of collagen model peptides. Protein Sci..

[26] Xu S.F., Xiao H.Z., Bi S. (2022). The improvement of dispersity, thermal stability and mechanical properties of collagen fibers by silane modification: An exploration for developing new leather making technology. J. Leather Sci. Eng..

